# Short-Term Salicylic Acid Treatment Affects Polyamine Metabolism Causing ROS–NO Imbalance in Tomato Roots

**DOI:** 10.3390/plants11131670

**Published:** 2022-06-24

**Authors:** Ágnes Szepesi, Péter Poór, László Bakacsy

**Affiliations:** Department of Plant Biology, Faculty of Science and Informatics, Institute of Biology, University of Szeged, Közép fasor 52, 6726 Szeged, Hungary; poorpeti@bio.u-szeged.hu (P.P.); bakacsy@outlook.com (L.B.)

**Keywords:** copper amine oxidase, nitric oxide, polyamines, root, salicylic acid, tomato

## Abstract

The phytohormone salicylic acid (SA) can influence the polyamine metabolism in plants. Additionally, polyamines (PAs) can regulate the synthesis of SA, providing an exciting interplay between them not only in plant growth and development but also in biotic or abiotic stress conditions. The effect of SA on polyamine metabolism of leaves is well-studied but the root responses are rarely investigated. In this study, tomato roots were used to investigate the effect of short-term exposition of SA in two different concentrations, a sublethal 0.1 mM and a lethal 1 mM. To explore the involvement of SA in regulating PAs in roots, the degradation of PAs was also determined. As both SA and PAs can induce reactive oxygen species (ROS) and nitric oxide (NO) production, the balance of ROS and NO was analyzed in root tips. The results showed that 0.1 mM SA induced the production of higher PAs, spermidine (Spd), and spermine (Spm), while 1 mM SA decreased the PA contents by activating degrading enzymes. Studying the ROS and NO levels in root tips, the ROS production was induced earlier than NO, consistent with all the investigated zones of roots. This study provides evidence for concentration-dependent rapid effects of SA treatments on polyamine metabolism causing an imbalance of ROS–NO in root tips.

## 1. Introduction

The phytohormone salicylic acid (SA) is known to induce multiple responses for plant growth and development, and also during abiotic and biotic stress conditions, its role is inevitable. Additionally, polyamines are essential for plant growth regulator compounds involved in multiple plant physiological responses from development to biotic and abiotic stress types. SA can interact with PAs, affecting the ROS and NO production, which strongly connects their metabolism [1,2]. Nowadays, studying *Arabidopsis thaliana* L., new evidence suggests that this interaction is more complex [3]. However, our knowledge is very limited on how SA can influence the root polyamine metabolism and what its role is in the ROS–NO interaction.

Biosynthesis of PAs positively affects the optimal PA metabolism in plants. It is well-studied that SA can activate the gene expression of PA synthesis genes, arginine decarboxylase (ADC), and ornithine decarboxylase (ODC) in different plants, e.g., maize (*Zea mays* L.) [4]. During drought stress, SA was reported to regulate biosynthesis of PAs in oat (*Avena sativa* L.) plants [5]. Additionally, in the leaves of Yali pear (*Pyrus bretschneider* cv. Yali), SA could regulate the biosynthesis of PAs after salt stress [6]. It is important to note that some evidence is contradictory, e.g., exogenous SA could increase PA levels in maize but this response decreased the drought stress tolerance of plants [4]. As our knowledge increases through new results about the involvement of SA in plant root growth and development [7,8], it is important to investigate the SA-induced responses in plant PA metabolism and decipher the potential connection between them in this organ.

PA degradation, which can also contribute to the fine-tuning of PA homeostasis, is catalyzed by two types of enzymes: copper amine oxidases (CuAOs) and flavin-containing polyamine oxidases (PAOs) [9]. Plant CuAOs are induced by SA, especially *AtCuAOY1* gene in *Arabidopsis thaliana* [10]. PAOs were also reported to be induced by SA after 24 h treatment in *Arabidopsis* [11]. Despite these results, root PA metabolism after short-term SA treatment is unexplored. In order to investigate the role of SA, we applied different concentrations of SA (0.1 and 1 mM) in short-term treatments to tomato roots.

## 2. Results

### 2.1. Effects of SA Treatments on Free Polyamines in Tomato Roots

**Figure 1:**
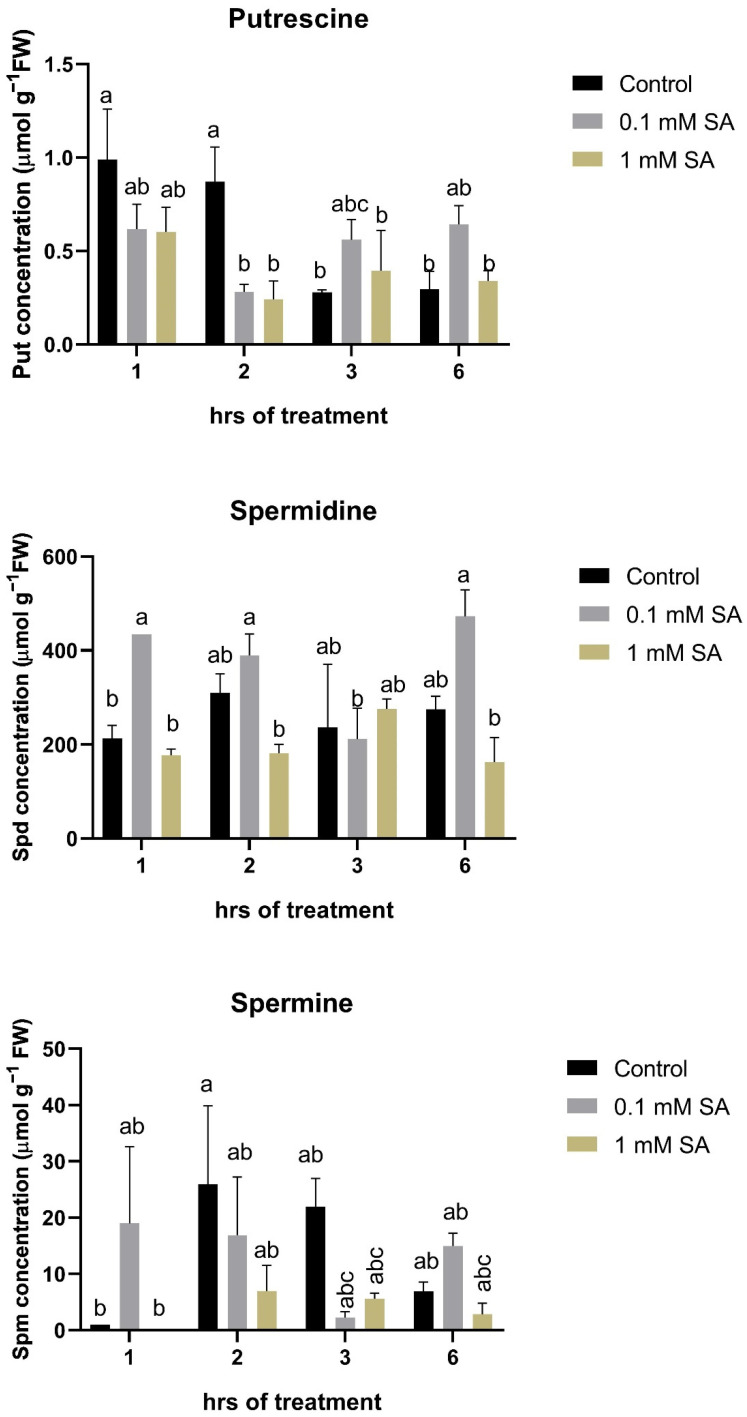
Changes in free polyamine (Put, Spd, and Spm) levels as a function of time in the roots of control or 0.1 mM or 1 mM SA-treated tomato plants. Error bars represent standard deviation (SD) of the means from three biological replicates. Different letters denote significant differences (one-way ANOVA, Tukey’s post hoc test, *p* < 0.05).

Both applied SA concentrations were effective to induce a decrease in Put level for 2 h, but after 2 h, 0.1 mM SA slightly induced its concentration, while 1 mM SA remained close to the control in the roots of tomato plants (Figure 1). In the case of Spd, 0.1 mM SA increased Spd levels in almost all time point except at 3 h, while 1 mM SA decreased Spd contents (Figure 1). At the same time, Spm levels did not change significantly after the applied SA concentrations during this time period (Figure 1).

### 2.2. Effects of SA Treatments on Total Polyamines and Ratio of (Spd + Spm)/Put

**Figure 2:**
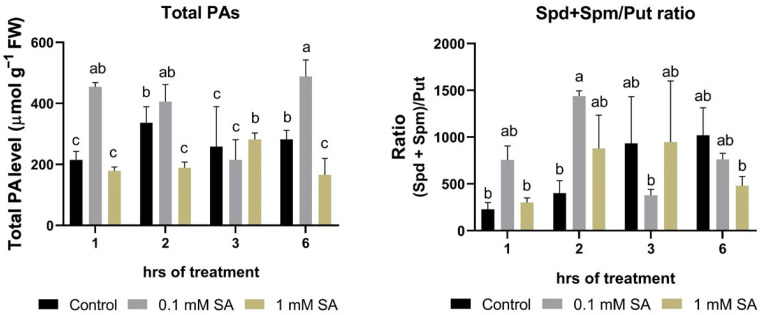
Changes in total polyamine (PA) levels and ratios of (Spd + Spm)/Put as a function of time in the roots of control or 0.1 mM or 1 mM SA-treated tomato plants. Error bars represent standard deviation (SD) of the means from three biological replicates. Different letters denote significant differences (one-way ANOVA, Tukey’s post hoc test, *p* < 0.05).

There were no significant differences between the total PA levels of the control and 1 mM SA-treated roots during the investigated time period (Figure 2). However, 0.1 mM SA showed a higher trend of total PAs except at 3 h, and this trend was reflected in the ratio of Spd + Spm/Put, showing that SA in this concentration could effectively induce the production of higher PAs in the roots of tomato plants (Figure 2).

### 2.3. Effects of SA Treatments on Polyamine Degradation Enzymes

**Figure 3:**
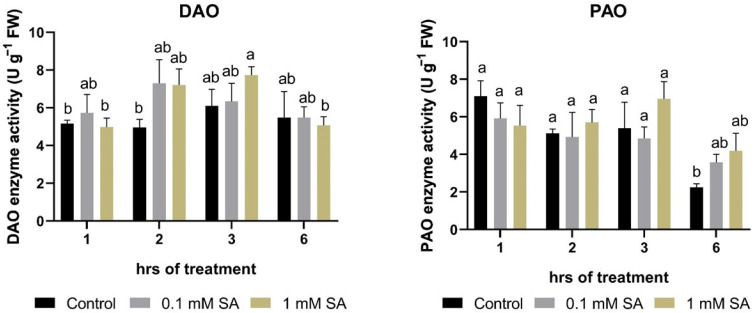
Changes in enzyme activities of copper amine oxidase (DAO) and polyamine oxidase (PAO) as a function of time in the roots of control or 0.1 mM or 1 mM SA-treated tomato plants. Error bars represent standard deviation (SD) of the means from three biological replicates. Different letters denote significant differences (one-way ANOVA, Tukey’s post hoc test, *p* < 0.05).

Both of the investigated PA-degrading enzyme activities, DAO and PAO, showed slight changes in roots, suggesting that this regulation type of PA levels could not be significantly involved in short-term responses to exogenous SA in this organ (Figure 3).

### 2.4. Effects of SA Treatments on ROS and NO Levels

**Figure 4:**
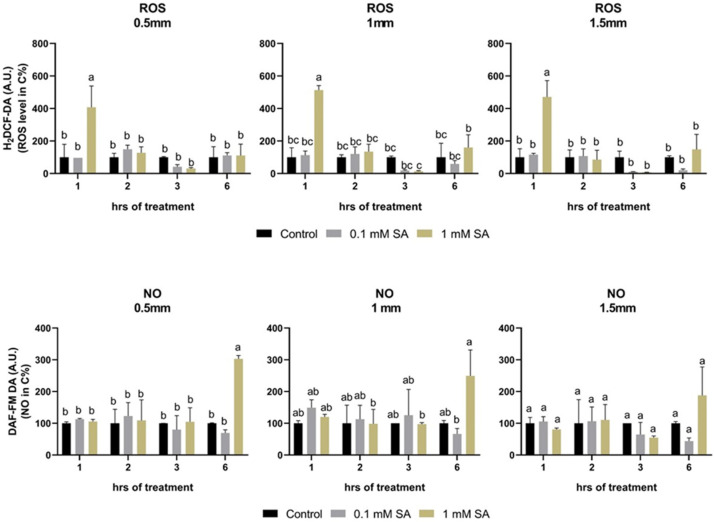
Changes in ROS and NO as a function of time in the root tips of control or 0.1 mM or 1 mM SA-treated tomato plants. Error bars represent standard deviation (SD) of the means from three biological replicates. Different letters denote significant differences (one-way ANOVA, Tukey’s post hoc test, *p* < 0.05).

Production of ROS and NO were analyzed in root tips at different distances from the apex of the root, namely 0.5, 1, and 1.5 mm. Observing these zones, ROS production was induced only at 1 h after 1 mM SA treatment but not after 0.1 mM SA treatment (Figure 4). However, NO production was inversely induced at 6 h only at 1 mM SA treatment (Figure 4).

## 3. Discussion

SA biosynthesis and signaling are very important in plant growth and development as well as under stress conditions [12,13]. While the leaf PA metabolism after SA treatment is well-studied, the SA-induced root PA metabolism responses are unknown. This prompted us to investigate the effect of SA treatment in two different concentrations, 0.1 mM as sublethal and 1 mM as lethal SA treatments, in tomato roots on PA metabolism and related balance of ROS–NO. In this study, we focused on short-term effects of SA on PA metabolism. These results revealed that the two concentrations of SA induced different changes in PA metabolism. While 0.1 mM SA induced the increase in free PAs, 1 mM SA decreased or did not change them compared to the control. There is new evidence that ADC2 can be a hub for hormone treatments, as it was shown that the expression of this gene was strongly induced not only after SA but some other hormones as well [11]. The only exception was at 3 h, where a slight difference could be seen, suggesting that this time point may be the change in the diurnal cycle as described by Gemperlova et al. (2006) [14] in tobacco plants. We investigated only the free PA levels, so the other forms of PAs, conjugated or bounded forms, which may also be important in SA-induced responses, remain to be investigated.

PA catabolism may be also an effective way to modulate the homeostasis of PAs [15]. In our study, the activities of PA-degrading enzymes did not show any significant alterations compared to control, suggesting that this short-term treatment did not induce changes in these enzyme activities; only slight fine-tuning or modulation occurred after SA treatments.

ROS and NO are also connected by PAs in different ways, e.g., during their synthesis or degradation [16]. Our study revealed that during this short-term treatment, only 1 mM SA could induce ROS production and NO production compared to control, but this induction was altered in time. ROS was produced earlier since NO increased only at 6 h after SA treatment. It is suggested that these alterations can contribute to the lethal effect of 1 mM SA in roots. Further investigations can clarify how this ROS–NO imbalance influences the late responses of tomato roots not only in growth but also stress conditions [17,18,19,20,21].

## 4. Materials and Methods

### 4.1. Plant Materials and Treatments

Tomato (*Solanum lycopersicum* L.) cultivar cv. Rio Fuego was used for experiments. Plants were grown hydroponically in a greenhouse of the Department of Plant Biology, University of Szeged as described by Szepesi et al. (2009) [22]. Germinated seeds were placed to perlite for 1 week and grown under 200 µmol m^−2^ s^−1^ photon flux density (F36W/GRO lamps, OSRAM SYLVANIA, Danvers, MA, USA), with 12/12 h light/dark period, day/night temperatures of 24/22 °C, and relative humidity of 55–60%. Plants were irrigated with modified Hoagland nutrient solution at pH 5.8. The experiments were replicated three times. Salicylic acid (SA) treatment was supplied by nutrient solution in different concentrations, 0.1 mM SA and 1 mM SA, for 6 h based on earlier experiments.

### 4.2. Analysis of Free Polyamine Levels by HPLC

For measuring the levels of free polyamines (Put, Spd, and Spm), high-performance liquid chromatography (HPLC) was used as described by Szepesi et al. (2022) [23]. Briefly, root samples were homogenized by 5% (*v/v*) perchloric acid. The homogenate was centrifuged at 4 °C for 10 min with 12,000 rpm by Eppendorf centrifuge (5424R, Eppendorf GMBH, Hamburg, Germany). The supernatant was used for analysis, and to neutralize, 2 M NaOH was pipetted to the supernatant. Polyamines were benzoylated by adding benzoyl chloride to produce benzoyl polyamine derivatives. Diethyl ether was pipetted to obtain the organic phase for drying. Dried samples were injected in acetonitrile into a JASCO HPLC System (JASCO, Tokyo, Japan). HPLC separation occurred by reverse-phase C18 column (250 × 4.6 mm internal diameter, 5 um particle size (Phenomenex, Torrance, CA, USA)). A UV–VIS detector (JASCO HPLC system, Japan) analyzed benzoyl polyamines at 254 nm wavelength. The mobile phase was ultrapure water/acetonitrile in a 55:45 (*v/v*) ratio, flow rate 0.5 mL min^−1^. Standards were Put, Spd, and Spm hydrochlorides from Sigma-Aldrich, Merck GMBH, Hamburg, Germany. The results were the means of three independent biological replicates expressed in µmol g^−1^ fresh weight^−1^.

### 4.3. Polyamine Catabolism: Diamine (DAO, EC 1.4.3.6) and Polyamine Oxidase (PAO, EC 1.4.3.4)

Copper amine oxidase (CuAO or DAO, EC 1.4.3.6) and polyamine oxidase (PAO, EC 1.4.3.4) activities were analyzed by a spectrophotometric method as described by Moschou et al. [17] with some modification. Tissues were ground in liquid N_2_ to fine powder, and an extraction buffer was added to each sample in a ratio of 1:3. The extraction buffer contained 0.2 M TRIS (hydroxymethyl)aminomethane (pH 8.0); 10% glycerol; 0.25% Triton X-100; 0.5 mM phenylmethanesulfonyl fluoride (PMSF); and 0.01 mM leupeptin. The homogenates were left on ice for 20 min and centrifuged for 10 min at 7000× *g* at 4 °C (Eppendorf centrifuge 5424R, Eppendorf GMBH, Germany). The reaction mixture contained supernatant and 100 mM potassium phosphate buffer (pH 6.6); then, the reaction was started by adding 1 M Put for DAO or 1 M Spd for PAO activity measurements. The reaction mixture was incubated for 1.5 h at 37 °C, and after, the reaction was stopped by adding 20% (*w*/*v*) trichloroacetic acid. To analyze the content of Δ1-pyrroline, a degradation product of enzymes, 2-aminobenzaldehyde (from 10 mg mL^−1^ stock solution) was pipetted to the reaction mixture. After centrifugation, absorbance of the supernatant was determined at 430 nm (KONTRON, Milano, Italy). The enzyme activity was expressed as the specific activity (U g^−1^ FW), where one unit (U) represents the amount of enzyme catalyzing the formation of 1 µmol of Δ1-pyrroline min^−1^.

### 4.4. Microscopic Analysis of Reactive Oxygen Species and Nitric Oxide in Tomato Root Tips

NO was detected with a specific fluorescent dye, 10 μM 4-amino-5-methylamino-2′,7′-difluorofluorescein (DAF-FM DA) (Sigma-Aldrich, St. Louis, MO, USA), and ROS was detected by using 10 μM 2,7-dichlorodihydrofluorescein diacetate (H_2_DC-FDA) (Sigma-Aldrich, St. Louis, MO, USA) as described by Gémes et al. (2011) [16]. The root tip sections were put on slides and covered with buffer and glass coverslip. Fluorescence intensity was detected with Zeiss Axiowert 200 M-type fluorescent microscope (Carl Zeiss Inc., Jena, Germany) equipped with an objective ×10. Digital photographs were taken from the samples with a high-resolution digital camera (Axiocam HR, HQ CCD camera; Carl Zeiss Inc., Jena, Germany) with a filter set 10 (excitation 450–495 nm, emission 515–565 nm). Fluorescence intensities (pixel intensity) of different zones (0.5, 1, and 1.5 mm distance from the tip) were measured on digital images within circular areas of 100 µm radii using Axiovision Rel. 4.8 software.

### 4.5. Statistical Analysis

Data presented here are the mean values from at least three independent experiments. Statistical analysis of two-way analysis of variance (ANOVA) was carried out with GraphPad Prism version 8.0.1.244 for Windows (GraphPad Software, La Jolla, CA, USA). Different letters on the bars denote significant differences (*p* < 0.05) based on Tukey’s post hoc test for multiple comparisons.

## 5. Conclusions

The concentration-dependent effect of SA on polyamine metabolism in plant roots was revealed in this study. A total of 0.1 mM SA as a sublethal concentration was effective to induce the production of higher PAS, while 1 mM SA as a lethal concentration decreased the PA contents. The ROS production increased earlier than NO production, showing a detrimental imbalance of these species in the roots. This study provides evidence for concentration-dependent effect of SA on polyamine metabolism causing an imbalance of ROS–NO in roots, helping our understanding of SA-induced PA metabolism in plants.

## Data Availability

Not applicable.
